# Moral Philosophy

**Published:** 1841-07-01

**Authors:** 


I.—MORAL PHILOSOPHY; OR THE DUTIES OF MAN CONSIDERED IN HIS INDIVIDUAL, DOMESTIC, AND SOCIAL RELATIONS. By *George Combe.* 8vo. Edinburgh and London, 1840, p. xvi. 440.

II.—NOTES ON THE UNITED STATES OF NORTH AMERICA, DURING A PHRENOLOGICAL VISIT, IN 1838-9-40. By *George Combe.* Three volumes, 8vo. Edinburgh and London, 1841, p. xxiv., 1260. With a Map and Illustrative Diagrams.

ALTHOUGH, on a cursory view, these works of Mr. Combe's may appear little calculated to embrace questions essentially, or even relatively, connected with the principles of medical philosophy; nevertheless, if candidly and considerately examined, his volumes will be found so full of natural and scientific truth, that the most experienced physician may derive from them a valuable and varied supply of excellent practical information. We say this much in their favour, without partiality or exaggeration; and, as a means of inducing others to follow our example, in studying his doctrines with an honest and independent spirit, we proceed to analyze such portions of our author's pages as seem best adapted to engage attention, in a medico-chirurgical journal.

### I. THE MORAL PHILOSOPHY.

Mr. Combe originally propounded his system of moral science in twenty lectures, delivered during the Winter sessions of 1835-6, to large classes of auditors by whom his instructions were received with an unusual degree of attention. While the course was in progress, they were successively reported, in the Edinburgh Chronicle, and thus an increasing interest was attracted to them, through the influence of this respectable journal. Soon afterwards, they were published in America, in the form of a convenient volume, and the whole impression was speedily exhausted in that country. During his recent sojourn there, the author republished his complete course, with suitable additions and corrections, and, since his return to Scotland in the early part of last year, he has subjected his entire doctrine to a particular and careful revision. It is, therefore, as an improved and matured edition, that his lectures on moral philosophy are now submitted to the public consideration and judgment.

Mr. Combe founds his moral philosophy on the phrenological principles —a circumstance which, he thinks, may appear to those who have not ascertained the truth and value of the latter science, to be nothing better than putting forth mere conjectures as the grounds of human duty; but, in harmony with the judgment of intuitive justice, he declares that the fact of many such persons withholding their assent to phrenology and its principles, does not necessarily render it an imaginary or hypothetical science; the denial of the circulation of the blood, by Harvey's cotemporaries, did not arrest the action of the heart, arteries, and veins; scientific truths exist independently of human observation and opinion. "As in general physiology and other sciences," he observes, "there are points in phrenology still unascertained, and these may hereafter prove to be important; but the future discovery of the functions of the spleen, will never overturn the ascertained functions of the lungs or spinal marrow; and, in like manner, the ascertainment of the uses of certain unknown parts at the base of the brain, will not alter the ascertained functions of the anterior lobe and coronal region. I consider the phrenological principles, on which I have founded the following lectures, to be established by such an extensive induction of facts, that they will sustain the severest scrutiny and not be found wanting; and I shall, with becoming resignation, abide by the verdict of those, who, by study and observation, shall have rendered themselves competent to judge of their merits."

On the present occasion, we select Mr. C.'s fourth lecture for exposition, as being most appropriate to our purpose of enlarging the sphere of prophylactic experience. It is intituled the "*Preservation of Bodily and Mental Health, a moral duty.*" In the first instance, however, as this author makes frequent allusion to the "physical and organic laws," and as it may be the reader's wish to know the precise meaning he attaches to these expressive terms, we introduce an explanation of their import according to the lecturer's usage. This definition of the acceptation in which he employs them, occurs in another philosophical work* of his, as in the following extract:—

"If the reader keep in view, that God is the creator; that Nature, in the general sense, means the world which He has made;—and, in a more limited

---

* The Constitution of Man, considered in relation to external objects; 8vo. London, 1835, p. 27, the third edition, revised, corrected, and enlarged.

sense, the particular constitution which he has bestowed on any special object, of which we may be treating; and that a law of Nature means, the established mode in which the actions and phenomena of any creature or object exhibit themselves, and the obligation thereby imposed on intelligent beings to attend to it,—he will be in no danger of misunderstanding my meaning. Every natural object, then, has received a definite constitution, in virtue of which it acts in a particular way. There must, therefore, be as many natural laws, as there are distinct modes of action of substances and beings, viewed by themselves. But substances and beings stand in certain relations to each other, and modify each other's action in an established and definite manner, according to that relationship; altitude, for instance, modifies the effect of heat upon water. There must, therefore, be also as many laws of nature, as there are *relations* between different substances and beings. It is impossible, in the present state of knowledge, to elucidate all these laws—numberless years may elapse before they shall be discovered; but we may investigate some of the most familiar and striking of them. Those that most readily present themselves bear reference to the great classes into which the objects around us may be divided, namely physical, organic, and intelligent.

1st. The *physical* laws embrace all the phenomena of mere matter: a heavy body, for instance, when unsupported, falls to the ground with a certain accelerating force, in proportion to the distance which it falls; and its own density; and this motion is said to take place according to the law of gravitation. An acid applied to a vegetable blue colour, converts it into red, and this is said to take place according to a chemical law.

2ndly. *Organised* substances and beings stand higher in the scale of creation, and have properties peculiar to themselves. They act, and are acted upon, in conformity with their constitution, and are therefore said to be subject to a peculiar set of laws, termed the *organic*. The distinguishing characteristic of this class of objects is, that the individuals of them derive their existence from other organized beings, are nourished by food, and go through a regular process of growth and decay. Vegetables and animals are the two great sub-divisions of it. The organic laws are different from the merely physical: a stone, for example, does not spring from a parent stone; it does not take food; it does not increase in vigour for a time, and then decay and suffer dissolution; all which processes characterize vegetables and animals. The organic laws are superior to the merely physical. A living man, or animal, may be placed in an oven, along with the carcass of a dead animal, and remain exposed to a heat which will completely bake the dead flesh, and yet come out alive, and not seriously injured. The dead flesh is mere physical matter, and its decomposition by the heat instantly commences; but the living animal is able, by its organic qualities, to counteract and resist, to a certain extent, that influence. The organic laws, therefore, mean the established modes according to which all phenomena connected with the production, health, growth, decay, and death, of vegetables and animals, take place. In the case of each animal or vegetable of the same kind, their action is always the same, in the same circumstances. Animals are the chief objects of my present observations.

3rdly. Intelligent beings stand yet higher in the scale than merely organized matter, and embrace all animals that have distinct consciousness, from the lowest of the inferior creatures up to man. The two great divisions of this class are *intelligent and animal*—and *intelligent and moral* creatures. The dog, horse, and elephant, for instance, belong to the former class, because they possess some degree of intelligence, and certain animal propensities, but no moral feelings; man belongs to the second, because he possesses all the three. These various faculties have received a definite constitution, and stand in determinate relationship to external objects: for example, a healthy palate cannot feel wormwood sweet, nor sugar bitter; a healthy eye cannot see a rod partly plunged in water straight—because the water so modifies the rays of light, as to give to the stick the appearance of being crooked; a healthy sentiment of benevolence cannot feel gratified with murder, nor a healthy conscientiousness with fraud. As, therefore, the mental faculties have received a precise constitution, have been placed in fixed and definite relations to external objects, and act regularly;—we speak of their acting according to rules or laws, and call these the moral and intellectual laws."

With these important distinctions held in view, let us now attend to Mr. Combe's philosophy in establishing the doctrine, that *prophylaxy*, or the preservation of bodily and mental health, is a moral duty. At the outset then, we find him stating didactically, that the causes of bad health result from infringement of the *organic* laws. We are instructed by universal experience—that, without health, man is unfit for the successful discharge of his duties: he is, therefore, under every obligation to apply his knowledge in preserving himself in a sound state both of body and mind. This is a self-evident maxim, and will not be gainsaid; but, while its truth is readily admitted, there are very many persons who remain grievously ignorant of the means how to carry it as a general injunction into effect. Let us see how the moralist exposes the "causes of bad health," by a plain array of facts and arguments.

He remarks, at p. 75, that, in tracing to their source the calamities which arise to families and individuals from bad health and untimely death, attended by deep laceration of the feelings and by numerous privations, it is surprising how many of these calamities may be discovered to result from slight but long continued deviations from the dictates of the organic laws; so slight that at first scarcely any injurious or even disagreeable effect was observed, but which gradually augmented until the most ruinous consequences were produced. Perhaps, he says for an instance, the victim was an ardent student; and, under the impulse of a laudable ambition to excel in his profession, he studied with so much intensity, and for such long periods in succession, that he overtasked his brain and destroyed his bodily health. Equally ignorant of the organic laws with himself, his parents and relations were rejoicing in his diligence, and forming fond expectations of the brilliant future that must, in their estimation, await one so gifted in virtuous feelings, in intellect, and in industry; when suddenly he was seized with fever, with inflammation, or with consumption; and, in a short time, he was carried to the tomb. On this melancholy picture, Mr. Combe pathetically but justly observes—the heart bleeds at the sight, and the ways of Providence seem hard to be reconciled with our natural feelings and expectations: yet, when we trace the catastrophe backwards to its first causes, it is ascertained to have had no mysterious origin. The very habits which appeared so amiable to the spectators, and so well calculated to lead to such exeellent attainments, were practically erroneous: and, indeed, there was not one link wanting to complete the connection between the fatal habits and the disastrous event which all so seriously lament. We press this picture on the gravest consideration of our readers, as available in their hands for the benefit of their youthful friends, of their own families, and perchance of themselves.

Another cause by which both health and life are frequently destroyed, is *occasional* reckless conduct, pursued in ignorance of the laws of the human constitution. Mr. Combe exemplifies this cause in the two following cases, which we transcribe.

*Case* I.—"A young man in a public office, after many months of sedentary occupation, went to the country on a shooting expedition, where he exhausted himself by muscular exertion, of which his previous habits had rendered him little capable; he went to bed feverish, and perspired much during the night; next day he came to Edinburgh, unprotected by a great coat, on the outside of a very early coach; his skin was chilled, the perspiration was checked, the blood received an undue determination to the interior vital organs, disease was excited in the lungs, and within a few weeks he was consigned to the grave."

*Case* II.—This is an interesting illustration of our moralist's prophylactic principles: it is the case, communicated in his own words, of a medical gentleman—*Dr. Robert Macnish*—who is well known in the literary world by his "Anatomy of Drunkenness," and other instructive publications; and who, since this account was written, has fallen a victim to an attack of fever. Addressing Mr. Combe, he says, "On four several occasions, I have nearly lost my life from infringing the organic laws. When a lad of fifteen, I brought on a brain fever (from excessive study) which nearly killed me; at the age of nineteen I had an attack of peritonitis, (inflammation of the lining membrane of the abdomen), occasioned by violent efforts in wrestling and leaping; and while in France, nine years ago, I was laid up with pneumonia (inflammation of the lungs) brought on by dissecting in the great galleries of La Pitie with my coat and hat off in the month of December, the windows next to me being constantly open; and, in 1829, I had a dreadful fever, occasioned by walking home from a party, at which I had been dancing, in an exceedingly cold morning, without a cloak or great coat. I was for four months on my back, and did not recover perfectly for more than eighteen months. All these evils were of my own creating, and arose from a foolish violation of laws which every sensible man ought to observe and regulate himself by. Indeed I have always thought—and your book confirms me more fully in the sentiment—that, by proper attention, crime and disease, and misery of every sort, could, in a much greater measure than is generally believed, be banished from the earth, and that the true method of doing so is to instruct people in the laws which govern their own frame."

Another principle held by Mr. Combe in his prophylactic system is this —"the great requisite of health consists in the preservation of *all* the leading organs of the body in a condition of regular and *proportionate* activity; to allow none to become too languid, and none too active." In his estimation, the result of this harmonious activity is a pleasing consciousness of existence experienced when the mind is withdrawn from all exciting objects and turned inwardly on its own feelings. He gives a portraiture of the quiet, delightful enjoyment which accompanies health, in the words of a philosophical friend, whose description appears to be admirable. It was the remark of this sage, that he never considered himself in complete health, except when he was able to place his feet firmly on the turf, with his hands hanging carelessly by his sides and his eyes wandering over space; and, thus circumstanced, to feel such agreeable sensations arising in his bodily frame, that he could raise his mind to Heaven and thank God that he was a living man.

We can hardly doubt of the Creator's having intended that the mere play of our bodily organs should yield us pleasure. It is probable too, that this is the chief gratification enjoyed by the inferior animals; and, although man has received the high gift of reason, it does not necessarily follow that he should be deprived of the delight which the organic nature is fairly calculated to afford. There is much instructive, though too often neglected, truth in the sentiments p. 78-9, which we record here with cheerfulness and approbation.

"How different is the enjoyment which I have described," Mr. C. remarks, "arising from the temperate, active, harmonious play of every bodily function,—from sensual pleasure, which results from the abuse of a few of our bodily appetites, and is followed by lasting pain; and yet so perverted are human notions, in consequence of ignorance and vicious habits, that thousands attach no idea to the phrase *bodily pleasure,* but sensual indulgence. The pleasurable feelings springing from health are delicate and refined; they are the reward and the support of virtue, and altogether incompatible with vicious gratification of the appetites. I am afraid that so widely do the habits of civilized life depart from the standard of nature, that this enjoyment is known in its full exquisiteness, to comparatively few. Too many of us, when we direct our attention to our bodily sensations, experience, instead of it, only feelings of discomfort, anxiety, and discontent, which make us fly to an external pursuit, that we may escape from ourselves. This undefined uneasiness is the result of slight but extensive derangement of the vital functions, and is the prelude of future disease. The causes of these uneasy feelings may be traced in our erroneous habits, occupations, and physical condition; and until society shall become so enlightened as to adopt extensive improvements in all these particulars, there is no prospect of their termination."

With regard to the advantages of cleanliness and exercise, as elements in his prophylactic discipline, our moralist adduces a variety of illustrations, which are not the less apt and applicable because they are homely. It is instructive, he observes, to compare with our own, the modes of life of the lower animals whose actions and habits are directly prompted and regulated by the Creator, by means of their instincts; because, in all circumstances in which our constitution closely resembles theirs, their conduct is really a lesson read to us by the All-Wise himself. In a state of nature, animals are remarkably cleanly in their habits: the feathered tribes dress their plumage, and wash themselves in the brooks: the domestic cat carefully preserves a clean, sleek, shining fur: the dog rolls himself on grass or straw: when grazing, the horse does the same: the sow is invariably clean, wherever it is possible for it to be so; its bad reputation arises from its master leaving it no sphere of existence except dunghills and other receptacles of filth. Again, in a state of nature, there has been imposed on the lower animals in acquiring their subsistance, a degree of labour which amounts to a regular exercise of their corporeal functions. At the same time, their food has been so adjusted to their constitutions, that they are well nourished, but very rarely rendered sick through surfeit or the bad quality of what they eat. *In a state of nature* then, the animals are impelled directly by the Creator to act in this manner; and, when we study their organization and see what is necessary to preserve it in health and enjoyment, we cannot fail to admire the wisdom and benevolence displayed in their natural economy and constitution.

Following up his enlightened views with the object of making them conducive to the welfare of moral and intelligent beings, our experienced prophylactist proceeds to expatiate on the duty and comfort of cleanliness, in these expressive terms:—

"Man differs from the brutes in this—that instead of blind instincts, he is furnished with reason, which enables him to study himself, the external world, and their mutual relations; and to pursue the conduct which these point out as beneficial. It is by examining the structure, modes of action, and objects, of the various parts of his constitution, that man discovers what his duties of performance and abstinence in regard to health, really are. This proposition may be illustrated in the following manner. The skin has innumerable pores and serves as an outlet for the waste particles of the body. The quantity of noxious matter excreted through these pores in twenty-four hours is, on the very lowest estimate, about twenty-four ounces. If the passage of this matter be obstructed so that it is retained in the body, the quality of the blood is deteriorated by its presence, and the general health, which greatly depends on the state of the blood, suffers. The nature of perspired matter is such, that it is apt, in consequence of the evaporation of its watery portion, to be condensed and clog the pores of the skin; and hence the necessity for washing the surface frequently, so as to keep the pores open and allow the perspiration to be freely performed. The clothing, moreover, must be so porous and clean, as readily to absorb and allow a passage to the matter perspired, otherwise the same result ensues as from the impurity of the skin, namely, the obstruction of the process of perspiration. Nor is this all. The skin is an absorbing as well as an excreting organ, so that foreign substances in contact with it are sucked into its pores and introduced into the blood. When cleanliness is neglected, therefore, the evil consequence is two-fold; first, the pores, as we have seen, are clogged, and the perspiration obstructed; and, secondly, part of the noxious matter left on the skin or clothing, is absorbed into the system, where it produces hurtful effects. From such an exposition of the structure and functions of the skin, the necessity for cleanliness of person and clothing becomes abundantly evident; and the corresponding duty of cleanliness is more likely to be performed by those who know the preceding details, than by persons who are impelled to performance by bare injunctions. In some parts of the East, ablution of the body is justly regarded as a duty of religion; but it needs not to be told how extensively this duty is neglected in our own country. When men become enlightened, a warm bath, once a week at least, will be considered one of the necessaries of life:—those who are in the habit of keeping their skin in a proper condition, by means of bathing and friction, will bear testimony to the increase of comfort and activity which is thus secured."

Instead of entering into a description of the bones, muscles, blood-vessels, nerves and brain, and a demonstration that the necessity of bodily and mental labour, of temperance, of attention to ventilation, clothing and lodging, and of many other salutary observances, is written by the finger of God in the frame-work of our bodies, Mr. C. limits this part of his lecture to two observations. 1. Exercise of the bones and muscles is labour, as he justly remarks; and labour, instead of being a curse to man, is a positive source of his well-being and enjoyment. It is only excessive labour that is painful; and, in a rightly ordered community, he believes there would be no necessity for inordinate exertion. 2. Exercise of the brain is mental activity or labour, and this is intellectual, or moral, or animal, according to the faculties which are employed. Mental inactivity, therefore, implies inactivity of the brain; and, as the brain is the fountain of nervous energy to the whole system, the punishment of neglecting its exercise is great and severe—namely, feelings of lassitude, uneasiness, fear and anxiety, vague desires, sleepless nights, and a general consciousness of discomfort, with incapacity to escape from suffering; all which poison life at its source, and render it thoroughly miserable. On the other hand, when combined, with due bodily exercise, well-regulated mental activity is rewarded with gay, joyous feelings, an inward alacrity to discharge all our duties, a good appetite, sound sleep, and a general sense of happiness that causes days and years to fleet away without leaving a trace of misery behind. It is equally indispensable to shun over-exertion, and undue or premature mental excitement; but, owing to the constitution of society, it is difficult to avoid, in our habitual conduct, one or other of the extremes of indolence or excessive exertion. Many persons, born to wealth, have few motives to engage in active employments: and such individuals, particularly females, often suffer grievously in their health and happiness from want of rational pursuits, calculated to excite and exercise their bodies and minds. Others, again, who do not get riches by inheritance, are tempted to overwork themselves in acquiring wealth and independence. An expensive style of living is so general as to be felt by many to be unavoidable; and, to support it, they labour so incessantly that almost no time remains for the cultivation of their moral and intellectual powers, and for that repose of mind and exercise of body which are indispensable to health. Hence arises indigestion and numerous cruel diseases; and many persons, even after they have succeeded in obtaining wealth, are tormented with uncomfortable and discontented feelings. Mr. Combe strengthens the foregoing observations with an extract from Dr. Caldwell's excellent work on "Physical Education," which we transcribe as especially meriting the profound attention of medical philosophers.

This highly talented and zealous philanthropist* explains, in some forcible remarks, "the tendency of the embroilment of party politics and religious differences to over-excite the brain and produce insanity, and also dyspepsy, which is more allied to insanity than is commonly supposed. So true is this, that the one is not unfrequently converted into the other, and often alternates with it. The lunatic is usually a dyspeptic during his lucid intervals; and complaints, which begin in some form of gastric derangement, turn, in many instances, to madness. Nor is this all. In families where mental derangement is hereditary, the members who escape that complaint are more than usually obnoxious to dyspepsia. It may be added, that dyspeptics and lunatics are relieved by the same modes of treatment, and that their maladies are induced, for the most part, by the same

---

* This eloquent and truly practical work was originally published at Boston in America, in 1834; and, with some additions by the author and editor, it was republished in Edinburgh with the title—Thoughts on Physical Education, and the True Mode of Improving the Condition of Man; and on the Study of the Greek and Latin Languages; by Charles Caldwell, M.D. Professor of the Institutes of Medicine and Clinical Practice in Transylvania University. With Notes by Robert Cox, and a Recommendatory Preface by George Combe; 12mo. Edinburgh, and London, 1836.

causes. The passions of grief, jealousy, anger, and envy, impair the digestive powers; and dyspepsia is often cured by abandoning care and business, and giving rest to the brain, It is chiefly for this reason that a visit to a watering-place is so beneficial. The agitations of commercial speculations and too eager pursuit of wealth, have the same effect with party politics and religious controversy in over-exciting the brain; and hence, in all probability, the inordinate extent of insanity and indigestion in Britain, and still more in the United States.

Two objections are generally urged against these admirable prophylactic rules (we are not dealing with therapeutics at present)—these, in Mr. C's words, are obviously, the dictates of reason. The *first* is—that persons, who are always taking care of their health, generally ruin it; their heads are filled with hypochondriacal fancies and alarms, and they become habitual valetudinarians. Mr. C. replies to this objection, p. 84, with these indisputable observations:—

"All such persons are already valetudinarians before they begin to experience the anxiety about their health here described; they are already nervous or dyspeptic, the victims of a morbid uneasiness of mind, which, for want of other objects, is at last directed towards the state of their health. They are essentially in the right, however, as to the main cause of their distress, for their anxiety certainly does proceed from disorder of their organic functions. Their chief error lies in this, that their care of health proceeds from an anxiety without knowledge, and leads to no beneficial result. They take quack medicines, or follow some foolish observances, instead of subjecting themselves patiently and perseveringly to a regimen in diet, and a regular course of exercise, amusement, and relaxation,—the remedies dictated by the organic laws. This last procedure alone is what I call taking care of health; and I have never seen any human being become an invalid, or a hypochondriac, from adopting it. On the contrary I have known many individuals who, in consequence of this rational obedience to the organic laws, have ceased to suffer under the maladies which previously attacked them."

The *second* objection is, that many persons live in sound health to a good old age; hence, it is the safest plan to follow their example, and act on all occasions as impulse prompts, never doubting that our health will take care of itself, if we follow this manly course. In reply to this too common misconception, Mr. C. observes, from manifest experience, that constitutions differ widely in the amount of their native energies, and consequently in the extent of wear and tear and bad treatment which they are able to sustain without being ruined; and that, for this reason, one individual may be comparatively little injured by a course of action which would prove fatal to another with a feebler frame.

Our moralist holds, for the grand principle of his philosophy—that the natural laws really admit of no exceptions,—and that specific causes, sufficient to account for the apparent exceptions, exist in every instance. In some of these supposed deviations from the rule, the individuals may have enjoyed a very robust constitution, which it was difficult to subvert: others may have indulged in excess only at intervals, passing an intermediate period in abstinence, and permitting the powers of nature to re-adjust themselves and recover their tone, before they committed a new debauch; others, again, may have led an extremely active life, passing much of their time in the open air—a mode of action which enables the constitution to withstand a greater extent of intemperance than it can resist with sedentary employment. Now, of one and all of these persons, Mr. C. affirms without hesitation—that, if they had obeyed the organic laws, they would have lived still longer and more happily than they did by infringing them. In the course of his own observations, and ours is in entire accordance with them, he has never known an individual proceed perseveringly in a course of intemperance, either sensual or mental—that is, who habitually over-tasked his stomach or his brain—who did not permanently ruin his health, usefulness and enjoyment. It has long been our confirmed opinion, that most of the evils of life proceed from ignorance, improvidence and profligacy.

On this head, Mr. Combe's last observation is remarkable for its truth and impressive simplicity: we would therefore bring it prominently under the attention of our readers, particularly the least experienced.

"With regard to health," he says, "Nature may be said to allow us to run an account-current with her, in which many small transgressions seem at the time to be followed by no penalty, when, in fact, they are all charged to the debit side of the account, and, after the lapse of years, are summed up and closed by a fearful balance against the transgressor. Do any of you know individuals, who, for twenty years, have persevered in frequent feastings, who all that time have been constant diners out or diners at home, or the soul of convivial meetings, prolonged into far advanced hours of the morning, and who have resisted every warning and admonition from friends, and proceeded in the confident belief that neither their health nor strength was impaired by such a course? Nature kept an account-current with such men. She had, at first, placed a strong constitution and vigorous health to their credit, and they had drawn on it day by day, believing that, because she did not instantly strike the balance against them and withdraw her blessing, she was keeping no note of their follies. But mark the close. At the end of twenty years, or less, you will find them dying of palsy, apoplexy, water in the chest, or some other disease clearly referable to their protracted intemperance; or, if they escape death, you will see them become walking shadows, the ghosts of their former selves—in short, the beacons set up by Nature to warn others that she does not in any instance permit her laws to be transgressed with impunity. If sedulous instruction in the laws of health, would not assist the reason and moral and religious feelings of such persons to curb their appetites, and avoid these consequences, they must be reckless indeed. At least, until this shall have been tried and failed, we should never despair, or consider their case and condition as beyond the reach of improvement."

By the admission of our benevolent prophylactist, the dangers arising to health, from improper social habits and arrangements, cannot be altogether avoided by the exertions of individuals acting singly in their separate spheres. He remarks emphatically, that the great precept of Christianity—WE MUST LOVE OUR NEIGHBOURS AS OURSELVES—is inscribed in every line of our constitution; and, in consequence, we must render our neighbours as moral and intelligent as ourselves, before we can reap the full reward of our own knowledge and attainments. He inforces this principle by a practical illustration, and then proceeds to show the reasonableness and necessity of its application in active life. We must, therefore, he declares, produce a general conviction among the constituent members of society, that Providence forbids that course of incessant action which obstructs the path of moral and intellectual improvement, and leads to mental anxiety and corporeal suffering. We must farther induce them, by a simultaneous movement, to apply an effectual remedy in a wiser and better distribution of the hours of labour, relaxation, and enjoyment. Every one of us can testify, he affirms, that this is *possible,* so far as the real, necessary, and advantageous business of the world is concerned; for, he continues, we perceive that, by a judicious arrangement of our time and our affairs, all *necessary business* may be compressed within many hours fewer than we now dedicate to that object, so as to allow us a reasonable space for mental cultivation, exercise and amusement. Mr. Combe considers eight hours a-day an ample allowance of time for business and labour: this would allow eight hours more for enjoyment, and eight for repose, a distribution that would cause life to flow more cheerfully, agreeably, and successfully, than it can do under our present system of ceaseless competition and toil. We fear, indeed we are certain, that this will be very unpalatable doctrine to our mammonian idolators, who, unfortunately, are too numerous and too influential; the day will come, however, when our posterity must succeed in gaining this boon for themselves and their families; and, of this also we feel well assured, that the "labouring classes" will owe the advent of this millennium, in a chief degree, to the zeal and knowledge of medical philanthropists.

We should fail of doing justice to the excellent prophylactical philosophy, which we have endeavoured to place before our readers, in the preceding exposition, were we to omit the author's deductive summary of his doctrine. On p. 90-1, it is recorded as follows:—

"It appears then," he concludes, "that the foregoing considerations, that the study and observance of the laws of health is a *moral* duty; this conduct being clearly revealed by our very constitution as the will of God, and being, moreover, necessary to the due discharge of all our other duties. We rarely hear from divines an exposition of the duty of preserving health, founded on our natural constitution; because they confine themselves to what the Scriptures contain. The Scriptures, in prescribing sobriety and temperance, moderation and activity, clearly coincide with the natural law on this subject; but we ought not to study the former to the exclusion of the latter; for, by learning the structure, functions, and relations of the human body, we are rendered more fully aware of the excellence of the scriptural precepts, and obtain new motives to observe them in our perception of the punishments by which, even in this world, the breach of them is visited. Why the exposition of the will of God, when strikingly written in the book of Nature, should be neglected by divines, is explicable only by the fact, that when the present standards of theology were framed, that book was sealed, and its contents were unknown. We cannot, therefore, justly blame our ancestors for the omission; but it is not too much to hope that modern divines may take courage and supply the deficiency. I believe that many of them are inclined to do so, but are afraid of giving offence to the people. By teaching the people to regard all natural institutions as divine, because they proceed from the Creator, this obstacle to improvement may, in time, be removed, and religion may be brought to lend her powerful aid in enforcing obedience to the natural laws."

Holding them to be closely connected with health, the author proceeds to consider the subject of amusements, regarding which much difference of opinion prevails. When we have no true philosophy of mind, he premises, this question becomes altogether inextricable, because every disputant ascribes to human nature those tendencies, either to virtue or vice, which suit his favourite theory. Philosophers, however, cannot make and unmake mental faculties and their organs, nor vary the functions and laws of action of these to suit different views, be they ever so ingenious. He next observes—that, by frequent and long continued action, every mental organ becomes fatigued, just as the muscles of the leg and arm grow weary by protracted exertion. Indeed, it cannot be conceived that the mind is susceptible of fatigue at all, except through the organs. We can comprehend how the fibres of the organ of tune may become exhausted by a constant repetition of the same action, and demand repose; but, he concludes impressively the idea of an immaterial spirit becoming weary, is altogether inconceivable. From this law of our constitution, therefore, it is plain that the all-good and all-wise Creator intended variety of employment to be essential to our welfare. Hence He has graciously bestowed on us a plurality of faculties, each having its own peculiar organ, so that some may rest, while others are actively employed. Now, among these various faculties and organs, there are several which appear obviously destined to contribute to our amusement; "a circumstance," as Addison has remarked, "which sufficiently shows us, that Providence did not design this world should be filled with murmurs and repinings, or that the heart of man should be involved in gloom and melancholy." Mr. Combe adds this illustration of his doctrine:—

"We have received a faculty of the ludicrous, which, when active, prompts us to laugh and to excite laughter in others. We have received organs of tune and time, which inspire us with the desire, and give us the talent, to produce music. Our organs of voluntary motion are so connected with these organs, that when we hear gay and vivacious music played in well marked time, we instinctively desire to dance; and when we survey the effect of dancing on our corporeal frame, we discover that it is admirably calculated to promote the circulation of the blood and nervous influence all over the body, and thereby to strengthen the limbs, the heart, the lungs, and the brain; in short, to invigorate the health, and to render the mind alert, cheerful, and happy. To such of my audience as have not studied anatomy and physiology, and who are ignorant of the functions of the brain, these propositions may appear to be mere words or theories; but to those who have made the structure, functions, relations, and adaptations of the various organs a subject of careful study and contemplation, I feel assured that they will appear in the light of truths. If such they are, our constitution proves that amusement has been kindly intended for us by the Creator, and that therefore, in itself, it must be not only harmless, but absolutely beneficial."

This most instructive lecture concludes with an admonition and rules to distinguish between the use and abuse of natural gifts; with a "line of demarcation" between the use and abuse of the stage and the fine arts; and with an exhortation to "try all things," to maintain virtue as well as knowledge, to discern it. If then, the Creator has constituted our minds and bodies to be benefited by amusements; has given us faculties specially destined to produce and enjoy amusement; and has assigned a sphere of use and abuse to these faculties as well as to others; it is clearly injudicious in the amiable, the virtuous, the charitable and the religious—in persons meriting our warmest sympathy and respect—to place themselves in an attitude of hostility, and of open and indiscriminate denunciation, against amusements founded on the laws of our nature. Instead of bringing all the weight of their moral and intellectual character to bear upon the improvement and beneficial application of these institutions, as it is obviously their duty both to God and to society to do, they fly from them as pestilential, and leave the direction of them exclusively to those whom they consider fitted only to abuse them. This, our philanthropist avers, is an example of piety and charity smitten with a moral paralysis through ignorance, and with a fatal cowardice through want of discipline.

While examining this prophylactic lecture, we have enjoyed an extreme satisfaction in contemplating the author's motives and object, his zeal and sincerity; and we would trust that the preceding sketches may prove instrumental in persuading many of our readers to investigate the principles on which his doctrine is founded. Should any one ever have entertained a doubt of its truth and value, as a medical principle, they will here find abundant reason for concluding that the PRESERVATION OF BODILY AND MENTAL HEALTH IS A MORAL DUTY.

We must defer our review of Dr. Combe's "Notes on the Americans" till our next.

